# Apolipoprotein E gene polymorphism and its effect on anthropometric measures in normoglycemic subjects and type 2 diabetes

**DOI:** 10.1186/2251-6581-11-18

**Published:** 2012-10-08

**Authors:** Ozra Tabatabaei-Malazy, Hossein Fakhrzadeh, Mostafa Qorbani, Parvin Amiri, Bagher Larijani, Javad Tavakkoly-Bazzaz, Mahsa M Amoli

**Affiliations:** 1 Endocrinology and Metabolism Research Center, Tehran University of Medical Sciences, Tehran, Iran; 2 Department of Epidemiology & Biostatistics, School of Public Health, Tehran University of Medical Sciences, Tehran, Iran; 3 Department of Medical Genetics, School of Medicine, Tehran University of Medical Sciences, Tehran, Iran

**Keywords:** Apolipoprotein E, Gender, Obesity, Diabetes mellitus, Lipid

## Abstract

**Background:**

Apolipoprotein E (apo E) plays a major role in lipid metabolism, obesity and accordingly in development of diabetes and coronary heart disease (CHD). Our main objective was to evaluate the association between apo E gene polymorphism with anthropometric measures.

**Methods:**

Participants were selected from zone 17 Tehran/Iran. We assessed height, weight, body mass index (BMI), waist circumference (WC), blood pressure, serum fasting blood sugar, total cholesterol and triglycerides. Genotyping for apo E gene polymorphism was carried out using PCR-RFLP technique.

**Results:**

Among total study population (n=311), 156 subjects were diabetic. The apo E3/E3 was the most common genotype in our population while E2 and E4 alleles had lower frequencies, respectively. After adjustment for diabetes, the apo E2 and E4 alleles were significantly associated with hypercholesterolemia and WC, respectively (p= 0.009, 0.034). This association was also related to sex and age. The probability of having abdominal obesity in E4 allele carriers was increased from 0.22 to 8.12 in women and to 3.08 in age ≥ 50 years.

**Conclusions:**

Apo E polymorphism had significant influences on WC and total cholesterol level in patients with type 2 diabetes. This study highlights the importance of lifestyle modifications which may be more beneficial in hypercholesterolemic women carriers of E2 and E4 alleles concomitant central obesity.

## Background

The human apo E is polymorphic and its genetic variation can affect its anti atherosclerotic effects [1]. The common variants of apo E polymorphism include E2, E3, and E4 allelic isoforms and six corresponding genotypes (E3/E3, E3/E4, E2/E3, E4/E4, E2/E4, and E2/E2, ranked from most to least common). Previous studies have shown that unlike the E3 allele, the E4 allele is associated with hypercholesterolemia, increased levels of LDL, HDL and atherosclerosis. Whereas E2 allele is related to increased plasma concentrations of triglyceride (TG) and lipoprotein remnants, decreased LDL [2,3], and pro- or anti atherogenic effects [1]. So, genetic polymorphism of apo E can contribute to the variation in lipoproteins concentration. The meta-analysis of 82 studies on lipid levels and 121 studies on coronary outcomes has identified a linear relationship of apo E gene polymorphism with both LDL levels and coronary risk to some extent. They showed that carriers of E2 allele have a 20% lower risk of coronary disease and E4 carriers have a slightly higher risk, compared with individuals with the E3/E3 genotype [4]. Similar results have been previously reported in other studies [3,5].

It has been suggested that the apolipoprotein E (apo E) gene polymorphism may be associated with predisposition to diabetic complications (both micro- and macrovascular complications). This association was shown between apo E gene polymorphism and diabetic nephropathy, but not for diabetic retinopathy in type 1 diabetes [6]. But in type 2 diabetes was found that E2 allele protects from macro- and microvascular complications and E4 allele tends to increase the risk for vascular complications [7]. As apo E plays an important role in lipid metabolism and on the other hand hypercholesterolemia is an independent risk factor for premature CHD, apo E may be an important risk determinant for CHD in diabetic and non diabetic subjects [8]. Also, it was shown that apo E polymorphism has different effects in diabetic and non-diabetic populations [9,10].

Recent evidence from both animal and human studies showed the important role of apo E gene in the development of obesity and insulin resistance [11]. Obesity is a worldwide health problem [12], and associated with several diseases such as type 2 diabetes mellitus (T2DM), hypertension and CHD [12]. In addition, epidemiological and population studies have established a direct correlation between obesity and development of cardiovascular disease [13]. The clustering of several clinical phenotypes including hypertension, dyslipidemia, obesity, and diabetes in the same subject may be due to a shared genetic background [14,15]. However, the importance of different risk factors for T2DM differs between ethnic populations [16]. In this study we aimed to determine the influence of apo E gene polymorphism on anthropometric measures in a group of Iranian with ethnic who had T2DM in comparison non-diabetes subjects.

## Methods

### Study population

Study subjects were recruited from zone 17 of Tehran/ Iran. The inclusion criteria were all of subjects who interest to enroll in the study. This study comprised of 311 subjects; 155 non-diabetics and 156 diabetics from the similar area. T2DM was diagnosed according to American Diabetes Association criteria [17]. After obtaining informed consent, a personal and demographic questionnaire was filled for each patient. Then height, weight, waist circumference (WC) and blood pressure were measured and recorded.

Body Mass Index (BMI) was calculated based on the following formula; body weight /square height (kg/m^2^). Obesity was defined as BMI ≥ 30 kg/m^2^ according to WHO recommendations [14]. In each subject (at standing position), WC was considered as the widest measure with a measuring tape between the margin of lower limb and crest of iliac, based on centimeter by the same investigator. Abdominal obesity was defined as a WC > 102 cm in men, and > 88 cm in women [18]. Systolic and diastolic blood pressures were measured in right arm at sitting position after 5 – 10 – min rest. Hypercholesterolemia and hypertriglyceridemia were defined as total cholesterol ≥ 200 and triglyceride ≥ 150 mg/dl based on definition of hyperlipidaemia in NCEP ATP III a [19].

The study was approved by the Ethics Committee of Tehran University of Medical Sciences of Iran.

### Laboratory methods

Levels of TG and cholesterol were tested for all our subjects using standard enzymatic methods (Pars Azmun, Iran). DNA was extracted from leukocytes using salting-out method [20]. Genomic DNA was amplified by PCR with the following primers F5’-TCCAA GGAGC TGCAG GCGGC GCA and R5’-GCCCC GGCCT GGTAC ACTGC CA yielding a 218-bp DNA fragment that spans both apo E polymorphic sites. Simultaneous digestion of the 218-bp amplified product by AflIII and HaeII enzymes yielded 145-bp, 168-bp, and 195-bp fragments corresponding to apo E3, E2, and E4, respectively.

### Statistical analysis

Strength of association between different variables and apo E gene polymorphism was estimated by Chi-square and logistic regression using SPSS software, version 11.5. P ≤ 0.05 was considered as significant in statistical analysis.

## Results

The age of the participants in the study was 25–65 years and mean age was 46 ± 13 years. In this study 32.2% of subjects were male and 67.8% were female.

### Baseline characteristics in diabetic and non-diabetic participants

The frequency of smokers, dyslipidemic, hypertensive, diabetic subjects, and past history of CHD or T2DM in the participants were 10.7, 55.5, 60.2, 50.2, 10 and 33%, respectively. Baseline characteristics of participants (n=311) are shown in Table 1. There was no significant difference in distribution of apo E gene polymorphism frequency in diabetics and nondiabetics (p= 0.414).

**Table 1:** Baseline characteristics in type 2 diabetic patients and non diabetic subjects

**Variable**	**T2DM (n=156)**	**Non diabetic subjects (n=155)**	**P Value**
**Sex (M/F) (n/%)**	46(14.8)/ 110(35.4)	54(17.4)/ 101(32.5)	0.333
**Apo (E3/E3)/ (E2/E3)/ (E4/E3) (n/%)**	113(38.2)/16(5.4)/15(5.1)	118(39.9)/23(7.8)/11(3.7)	0.414
**Age (yr)**	49 ± 11	41 ± 13.7	<0.001*
**BMI (kg/m**^**2**^**)**	32.6 ± 4.6	25.7 ± 3.7	<0.001*
**SBP (mmHg)**	140 ± 24	124 ± 21	<0.001*
**DBP (mmHg)**	89 ± 13	80 ± 13	<0.001*
**WC (cm)**	97 ± 12	91 ± 13	<0.001*
**FBS (mg/dl)**	115 ± 60	94 ± 49	0.001*
**TChol (mg/dl)**	210 ± 48	202 ± 42	0.140
**£TG (mg/dl)**	240 ± 176	176 ± 151	<0.001*

**Legend: T2DM:** Type 2 Diabetes Mellitus, **M/F:** Male/Female, **BMI**: Body Mass Index, **SBP**: Systolic Blood Pressure, **DBP**: Diastolic.

Blood Pressure, **WC**: Waist Circumference, **FBS**: Fasting Blood Sugar, **TChol:** Total Cholesterol, **TG:** Triglyceride.

*P≤0.05 was considered as significant in statistical analysis, T- test.

**£** Analytic test was Mann–Whitney Test.

### Distribution of apo E genotype frequencies and mean level of anthropometric measures in diabetic and non-diabetic participants

The frequency of different polymorphism of apo E gene in our population is shown in Table 2. The mean level of anthropometric measures in subgroup of abnormal WC of diabetics and nondiabetics was significantly higher than in subgroup of normal WC which is shown in Table 2.

**Table 2:** Distribution of apo E genotype frequencies in type 2 diabetic patients and non diabetic subjects

**Variable**	**T2DM (n=156)**		**P Value**	**Non diabetic subjects (n=155)**		**P Value**
	**WC normal (n=51)**	**WC abnormal (n=105)**		**WC normal (n=81)**	**WC abnormal (n=73)**	
**Sex (M/F) (n/%)**	29(18.6)/ 22(14.1)	17(10.9)/ 88(56.4)	<0.001*	40(26)/ 41(26.6)	14(9.1)/ 59(38.3)	<0.001*
**Apo (E3/E3)/(E2/E3)/(E4/E3) (n/%)**	29(20.1)/5(3.5)/10(6.9)	84(58.3)/11(7.6)/5(3.5)	0.005*	62(41.1)/11(7.3)/6(4)	56(37.1)/11(7.3)/5(3.3)	0.965
**Age (yr)**	52 ± 12	51 ± 10	0.866	34 ± 10	46 ± 12	<0.001*
**BMI (kg/m**^**2**^**)**	25.4 ± 3	32 ± 5	<0.001*	26 ± 4	33 ± 4	<0.001*
**SBP (mmHg)**	131 ± 20	143 ± 25	0.003*	119 ± 22	135 ± 21	<0.001*
**DBP (mmHg)**	84 ± 13	90 ± 14	0.007*	78 ± 13	88 ± 13	<0.001*
**FBS (mg/dl)**	125 ± 68	140 ± 67	0.175	75 ± 8	80 ± 10	0.002*
**TChol (mg/dl)**	199 ± 46	215 ± 48	0.042*	188 ± 38	219 ± 42	<0.001*
**£TG (mg/dl)**	241 ± 211	269 ± 197	0.029*	135 ± 72	199 ± 133	<0.001*

**Legend: T2DM:** Type 2 Diabetes Mellitus, **WC**: Waist Circumference, waist circumference is >88 cm in women and >102 cm in men is defined as abnormal, **M/F:** Male/Female, **BMI**: Body Mass Index, **SBP**: Systolic Blood Pressure, **DBP**: Diastolic Blood Pressure, **FBS**: Fasting Blood Sugar, **TChol:** Total Cholesterol, **TG:** Triglyceride.

* P≤0.05 was considered as significant in statistical analysis, T- test.

**£££Ê£** Analytic test was Mann–Whitney Test.

### Apo E gene polymorphism association with anthropometric measures in diabetics & non-diabetics

Coefficients of univariate and multivariate regression models between independents variables (sex, age ≥ 50 years, apo E2/E3 or apo E4/E3 polymorphism versus apo E3/E3, and T2DM) and dependent variables (BMI ≥ 30, central obesity, high blood pressure, high level of total cholesterol or triglycerides) are shown in Table 3.

**Table 3:** Coefficients of univariate and multivariate regression models between independents variables and obesity, abdominal obesity, hypertension, high levels of total cholesterol and triglyceride

**Variable**	**Obesity (Y/N)**		**Abdominal obesity (Y/N)**		**Hypertension (Y/N)**		**Hypercholesterolemia (Y/N)**		**Hypertriglyceridemia (Y/N)**	
	**Univariate**	**Multivariate**	**Univariate**	**Multivariate**	**Univariate**	**Multivariate**	**Univariate**	**Multivariate**	**Univariate**	**Multivariate**
	**Odds Ratio (CI 95%)**	**Odds Ratio (CI 95%)**	**Odds Ratio (CI 95%)**	**Odds Ratio (CI 95%)**	**Odds Ratio (CI 95%)**	**Odds Ratio (CI 95%)**	**Odds Ratio (CI 95%)**	**Odds Ratio (CI 95%)**	**Odds Ratio (CI 95%)**	**Odds Ratio (CI 95%)**
**Sex (Female/Male)**	2.77*	3.07**	5.19*	8.12**	1.02	1.02	2.05*	2.10**	1.14	1.06
	(1.67-4.58)	(1.77-5.31)	(3.10-8.70)	(4.29-15.36)	(0.63-1.64)	(0.58-1.79)	(1.26-3.32)	(1.23-3.61)	(0.70-1.86)	(0.61-1.86)
**Age (>50/<50 yr)**	1.26	1.47	2.55*	3.08**	4.41*	3.26**	2.21*	2.05**	1.54*	0.91
	(0.79-2.01)	(0.87-2.51)	(1.57-4.16)	(1.64-5.78)	(2.66-7.31)	(1.86-5.72)	(1.37-3.57)	(1.19-3.51)	(0.96-2.46)	(0.52-1.58)
**Apo (E2/E3)/apo (E3/E3)**	0.85	0.83	0.89	0.99	0.98	1.08	0.42*	0.36**	1.16	1.12
	(0.43-1.68)	(0.40-1.71)	(0.45-1.79)	(0.44-2.24)	(0.49-1.96)	(0.51-2.29)	(0.21-0.84)	(0.17-0.77)	(0.58-2.30)	(0.52-2.38)
**Apo (E4/E3)/apo (E3/E3)**	0.73	0.59	0.41*	0.22**	1.09	0.88	1.19	1.03	2.04*	1.96
	(0.32-1.65)	(0.25-1.38)	(0.18-0.93)	(0.08-0.56)	(0.48-2.52)	(0.36-2.15)	(0.52-2.73)	(0.44-2.44)	(0.90-4.65)	(0.81-4.74)

**Legend: Obesity:** BMI≥30 kg/m^2^, **Abdominal obesity:** waist circumference is >88 cm in women and >102 cm in men, **Hypertension:** blood pressure is>130/85 mmHg.

**Hypercholesterolemia:** Total cholesterol is >200 mg/dl, **Hypertriglyceridemia:** Triglyceride is >150 mg/dl, **Y/N:** yes/no, **CI 95%:** confidence interval 95%.

* P < 0.2 in binary regression with univariate model is considered for analysis by binary regression with multivariate model. On the other hand P>0.2 in univariate analysis can. not be considered for analysis with multivariate model and are determined as NS.

* * P ≤ 0.05 in binary regression with multivariate model is considered as significant.

*BMI:* The influence of sex on BMI ≥ 30 was independent of diabetes or apo E gene polymorphism. *Central obesity:* Sex, aging, and apo E4 allele had significant influence on central obesity which these effects were dependent to diabetes.

*Hypertension:* Aging had a significant influence on hypertension dependent to diabetes.

*Hypercholesterolemia:* Sex, aging, and apo E2 allele had significant influence on hypercholesterolemia which these effects were independent of diabetes.

*Hypertriglyceridemia:* We didn’t find any significant association between independent variables and hypertriglyceridemia.

The details of all above mentioned results are shown in Table 3.

## Discussion

The complex polygenic diseases such as diabetes could be complicated by phenomena such as variable age at onset disease, or environmental triggers [21]. Although, some phenotypes such as BMI and insulin resistance have been affected largely by genetic factors, others such as abdominal obesity have been affected largely by non-genetic factors, probably environmental [22,23]. So, assessment characteristics of well defined phenotypic abnormalities could show clearly the association between genome and environment. On the other word by assessment the association between apo E gene polymorphism and anthropometric measures as risk factors of diabetes, it is possible to determine the association between apo E gene polymorphism and diabetes.

The role of apo E gene polymorphism in anthropometric measures has been studied in different populations. Although this study is conducted in a small sample size, this is the first study on association between apo E gene polymorphism and anthropometric measures in Iranian population. In this study Apo E3 was the most common allele in our population, and E2 followed by E4 allele have been found less frequent both in diabetics and nondiabetics. These findings are in agreement with previous reports [10,24]. So, the frequency of apo E polymorphism could be reasonable different according to environmental factors and lifestyle behaviors, obesity, gender, population related differences (such as geographical differences), presence of CHD, and even undertreatment lipid lowering drugs [25-31].

In subgroup of patients with abnormal WC in our study, all components of anthropometric measures were higher than subgroup that had normal WC. Previous evidence also supports our finding [32-34] which may be related to insulin resistance. Our study was shown a significant difference in distribution of apo E allele frequency in patients stratified according to WC only in diabetic group. Also, there was a significant influence for apo E4 allele on central obesity. But this effect was nonsignificant for apo E2 allele. Oh et al. [26] found a significant correlation between WC and apo E4 allele in women with a family history of diabetes which was independent of hyperlipidemia. This suggests that mechanisms other than lipid pathways might be involved in apo E action [35]. As the weight control is important for improving insulin sensitivity and reducing CVD risk, therefore it might be speculated that in apo E4 carriers weight control play crucial role in control of hyperglycemia and reducing CVD risk.

In our study, apo E2 allele was significantly associated with hypercholesterolemia both in women and in patients aged > 50 which was independent of diabetes. Also, lipid levels did not significantly differ between subjects with apo E3/E3 and apo E4/E3. This is in agreement with our previous results and also results by Pedro-Botet et al. who found significantly higher level of total cholesterol in patients with E2 isoform [24,29]. In contrast, Oh et al. [26] found that in men with family history of diabetes, apo E2 and also apo E4 carriers had higher level of total and LDL cholesterol compared to those with apo E3/E3, although the difference was not significant. In addition, they have found that in both sexes with no family history of diabetes, apo E2 was associated with normal levels of total and LDL cholesterol [26]. It seems the effect of apo E polymorphism on lipid metabolism may vary according to genetic background. However, the apo E polymorphism especially apo E4 seems to be a risk factor for CHD development independent of association with high level of total and LDL cholesterol [35-39].

An association between apo E2 allele and hypertriglyceridemia has been consistently reported in healthy populations [40-43]. Dallongeville et al. in their meta-analyzes have shown that triglyceride concentration is significantly higher in carrier of E2 or E4 allele than in carriers of E3 allele, at least among men and also the presence of E2 allele was related to lower and the E4 allele to higher level of total and LDL cholesterol in plasma relative to those with the E3 allele [37]. Although our abnormal WC subgroup of T2DM patients had higher triglycerides level than the diabetics with normal WC, there were no significant correlation between apo E gene polymorphism and triglycerides level in multivariate regression. This finding is in agreement with most studies that have been done in European diabetic patients [10,44,45], but it is in contrast to studies on diabetic patients among Asian populations [46-48] which higher level of triglycerides in carriers of E2 allele have been reported.

In our study, the probability of being obese (BMI ≥ 30) was increased significantly in women, which was independent of apo E polymorphism or diabetes. This parameter is known to influence lipid metabolism or insulin resistance and may interfere with genetic factors. The influence of lifestyle on apo E polymorphism was clearly shown in two parts of Belgium [49]. Although apo E has long been known as athero-protective and in excess of circulating lipids apo E would be expressed as a key peripheral contributor to the development of obesity and related metabolic dysfunctions. There are some reports in contrast to our finding. Data from the Atherosclerosis Risk in Communities (ARIC) study, showed that apo E genotypes were associated with BMI in the order of apo E4 < apo E3 < apo E2 [50]. Another epidemiological study showed that, in older women with a family history of diabetes, apo E4 /E 4 and apoE3/E4 genotypes were correlated with increased waist circumference and obesity [26].

In this study, we observed that there were sex-specific effects on anthropometric measures. For example, abnormal WC was more prevalent in females both in diabetics and nondiabetics. Also, our data has shown that the probability of having obesity, central obesity, and hypercholesterolemia are increased significantly in diabetic women independent of apo E polymorphism. The sex-specific distribution of apo E gene was not differing significantly between two groups. This might be as a result of deviation in female/male ratio in our population. However, gender related differences are difficult to determine due to variations of hormonal status during pre- and post menopause in women and their effects on plasma lipid profile [51].

In our patients the probability of having central obesity, hypertension and hypercholesterolemia was increased significantly in diabetics according to age (≥ 50 years). When we assessed the effect of apo E2 allele on hypercholesterolemia, this trend was equal 0.36 which by considering the effect of age, this figure was changed to 2.05. The trend for increasing central obesity in E4 allele carriers was 0.22 without considering the effect of age and changed to 3.08 in presence of age > 50 years. These results showed the influence of apo E polymorphism on abdominal obesity and hypercholesterolemia was significantly changed in presence age > 50 years. One study was evaluated this effect on postprandial triglycerides [52]. They found a significant interaction between age and apo E on postprandial triglycerides. In their study was unmasked the unfavorable effect of E2 and E4 alleles on under curve concentration by aging.

As it is expected, we found a significant increase in blood pressure just at the presence of diabetes and aging. A positive correlation between total serum cholesterol level and blood pressure [53] had been shown in population-based studies; which might imply the effect of apo E genotype [54]. However, we didn’t find any significant association between apo E genotype and hypertension in our study which could be also due to the variation in sex distribution.

In this study, there was no information about diet of our patients therefore we don’t have any precise explanation for these differences. Because our study was carried out in a small population, therefore the effects observed might not be subject to generalization. The possible contribution of such association to the observed relationship between apo E4 and abdominal obesity also remains to be clarified by prospective studies.

## Conclusion

In summary, the present study shows low frequency of the E2 and E4 alleles in our study population and indicates that apo E polymorphism might play a role in determining plasma lipids levels. The significant inter-relation between the apo E polymorphism, abdominal obesity, and change in total cholesterol levels supports epidemiological findings.

Although lifestyle modification is beneficial in all people, it may be especially recommendable in women carriers of E2 allele to modify potentially elevated fasting cholesterol, and also in carriers of E4 allele to control weight. In addition, apo E4 allele might be a predictor for CHD in diabetics by influencing central obesity. Our data suggest that this risk factor probably exert its atherogenic effects through diverse mechanisms.

## Endnote

^a^National Cholesterol Education Program (Adult Treatment panel III).

## Competing interests

The authors had no conflict of interest.

## Authors’ contributions

OT-M conceived of the study, wrote draft the manuscript and performed the statistical analysis. HF conceived of the study, and participated in its design and coordination. MQ performed the statistical analysis. PA carried out the molecular genetic assessment. BL conceived of the study. JT-B conceived of the study. MMA conceived of the study, carried out the molecular genetic assessment and helped to draft the manuscript. All authors read and approved the final manuscript.

## Financial support

This study was performed in Endocrinology & Metabolism Research Center of Tehran Universityof Medical Sciences without financial support.
